# End-to-end modeling of fuel injection via static coupling of internal flow and ensuing spray

**DOI:** 10.1038/s44172-022-00038-z

**Published:** 2022-12-09

**Authors:** Roberto Torelli, Yuanjiang Pei, Yu Zhang, Sibendu Som

**Affiliations:** 1 Transportation and Power Systems Division, Argonne National Laboratory, 9700 South Cass Avenue, Lemont, IL 60439 USA; 2 Aramco Americas: Aramco Research Center – Detroit, 46535 Peary Court, Novi, MI 48377 USA

**Keywords:** Mechanical engineering, Computational science, Fluid dynamics

## Abstract

Accurate knowledge of fuel spray behavior is of utmost importance for liquid-fuel-based combustion systems. Fuel properties, injector geometry, operating conditions, and thermal state of the combustion chamber determine the fuel’s ability to mix and burn efficiently. Three-dimensional computational-fluid-dynamics models can reveal the complex dynamics of the injector’s internal flow, as well as the spray breakup, evaporation, mixing, and combustion. However, time and length scales of in-nozzle flow and ensuing spray can differ by several orders of magnitude, limiting the feasibility of a simultaneous solution of the entire chain of physics. This work explores an end-to-end approach to decouple the problem at the injector outlet via a static-coupling framework. Flowfields are sampled at the injector exit, stored into spatiotemporally resolved maps, and used to initialize a Lagrangian spray whose properties reflect the flow’s instantaneous state as predicted by the in-nozzle flow simulations. Comparisons against typical rate-of-injection results and qualitative validation against optical spray data highlighted the ability of static coupling to unveil spray physics that would otherwise be missed.

## Abstract (web summary)

Roberto Torelli and colleagues propose a numerical framework to characterize fuel injection in internal combustion engines at multiple length and time scales. The approach demonstrates potential for increased fidelity in the flow dynamics by means of an affordable end-to-end methodology that links realistic injection operation to fuel combustion and engine emissions.

## Introduction

Fuel sprays find many applications in transportation. Some examples are aerospace, marine, rail, and automotive, to name a few. Hence, the fuel injection system is usually regarded as an integral part of any propulsion unit that is powered by gaseous or liquid fuels, whether this unit is an aircraft’s turbojet or a passenger car’s piston engine.

With a focus on automotive and transportation, it is well known that these industries are currently undergoing a revolutionary and transformational phase to address undeniably needed and tightening criteria pollutants and CO_2_ emissions regulations. Due to their zero tank-to-tailpipe emissions signature, fully electrified propulsion technologies (e.g., battery, fuel cell) are challenging well-established conventional technologies such as gasoline- and diesel-powered internal combustion engines (ICEs)^1^. On the other hand, current estimates suggest that ICEs will still play a major role in the transportation industry during the next three decades as the energy supply landscape evolves progressively^2^, either as part of electrified hybrid systems for light- and medium-duty applications or as the main propulsion source for on-road and non-road heavy-duty commercial vehicles, for which the deployment of fully electrified propulsion system technologies on a large commercial scale might still not happen for decades.

Therefore, in an effort to minimize emissions without incurring excessive cost and complexity for exhaust aftertreatment devices, modern ICEs must achieve the highest fuel efficiency and cleanest combustion during all engine cycles and for all operating conditions (e.g., idle, partial and high loads, cold start, etc.). Within this framework, injection systems are a fundamental piece of the equation as they determine the condition of the fuel jets entering the engine’s cylinders. Such condition impacts the evolution and characteristics of the ensuing fuel spray, in turn determining the fuel’s ability to mix with the surrounding oxidizer adequately and burn efficiently. Researchers and engineers rely on a combination of experiments and simulations to test new injector designs and select the most promising ones. Experiments can provide information that range from simple injector characterizations of mass flow rate and discharge coefficient to sophisticated non-destructive diagnostics that can return spatially and temporally resolved information about the internal geometry and the motion of the moving parts of an injector such as the injector’s needle valve. This information allows researchers to build three-dimensional (3-D) computational fluid dynamics (CFD) models of the injectors, which have become increasingly important during the research and development phase of these components. Indeed, when supported with experimental validation data, high-fidelity CFD models can provide an invaluable understanding of the physics and the behavior of the flow within and immediately outside the injector. These models allow to isolate the effect of fuel properties (e.g., density, viscosity, and saturation pressure), identify a fuel’s propensity to exhibit undesirable behaviors such as fuel cavitation, and perform time-resolved evaluations of the injector performance under wide ranges of operating conditions. This acquired knowledge can consequently enable researchers and engineers to make informed design choices and screen multiple solutions without incurring in the cost of building physical prototypes for all tested designs.

The ability of these CFD models to predict the internal flow behavior has been validated against X-ray measurements such as those reported by Tekawade et al.^3^ and has been documented in the work of Guo et al.^4^. However, the description of the ensuing spray rarely relies on the detailed knowledge that can be gained via the internal nozzle flow simulations described so far. The typical modeling approach used to initialize the ensuing fuel spray generally relies on a Lagrangian-Eulerian (L-E) formulation in which the liquid spray originating at the injector’s orifice exits is tracked in a Lagrangian fashion while the surrounding gas phase is modeled with a typical Eulerian approach. Furthermore, the introduction of the Lagrangian spray, represented by statistical entities known as parcels, is commonly based on the rate of injection (ROI) methodology. As described in the Methods section, the ROI approach is intrinsically limited by its one-dimensional nature and minimal ability to impress physics-based, spatiotemporal variations to the behavior of the spray.

In this work, an alternative end-to-end methodology, based on the static coupling of the fully Eulerian internal flow and the ensuing Lagrangian spray, is explored to highlight the potential benefits of the static-coupling approach as opposed to the typical ROI one. As reported in greater detail in the Methods section, the static-coupling approach, also known as one-way coupling, was applied to an eight-hole heavy-duty (HD) diesel injector operating with a gasoline-like fuel under a gasoline compression ignition combustion mode. A time-set of two-dimensional injection maps was generated at runtime while simulating the internal flow. The maps were extracted on planar cross-sections located at the exit of each of the injector’s eight orifices and stored in a map file that was subsequently used as input to the L-E spray simulations. The information contained in the maps (e.g., flow velocity vectors, liquid volume fraction, temperature distributions, etc.) was then used to initialize Lagrangian spray parcels according to the discrete droplet modeling proposed by Dukowicz^5^ and briefly described in the Methods section. The analysis presented in the Results section compares the results obtained with the two methodologies, i.e., ROI and one-way coupling, highlighting the differences that arise in the predicted spray behavior and morphology when the latter approach is used, as well as their impact on the combustion behavior and emission signature of the engine. It is shown that the proposed end-to-end framework is able to link the internal flow behavior to the ensuing spray and unveil the presence of orifice-to-orifice variability observed in optical spray experiments, as reported in the Results section. In addition, as the one-way coupling maps are space- and time-resolved, this static coupling approach also provides the ability to capture spatiotemporal changes that cannot be modeled with the ROI approach in a predictive way. Indeed, one would need well-resolved optical experiments (in both space and time) to characterize the temporally varying spray behaviors that can be expected due to the complex fluid dynamics of the internal nozzle flow. Even with such availability of experimental data, one would also need to impose this time-varying profiles as a predefined boundary condition, defying the goal of using a spray model that is predictive in representing the dynamics of the spray itself. This work shows how using an end-to-end framework such as the one presented here allows to overcome this barrier and provides an estimate of the impact that the injector behavior can have on the ensuing spray as well as the fuel mixing, combustion, and emission signature of the ICE. It is noted that all the findings presented in this work are only supported by qualitative comparisons with optical spray experiments and that further experimental work and analyses are required to achieve formal quantitative validation.

## Results

### Injection operating conditions

**Fig. 1:**
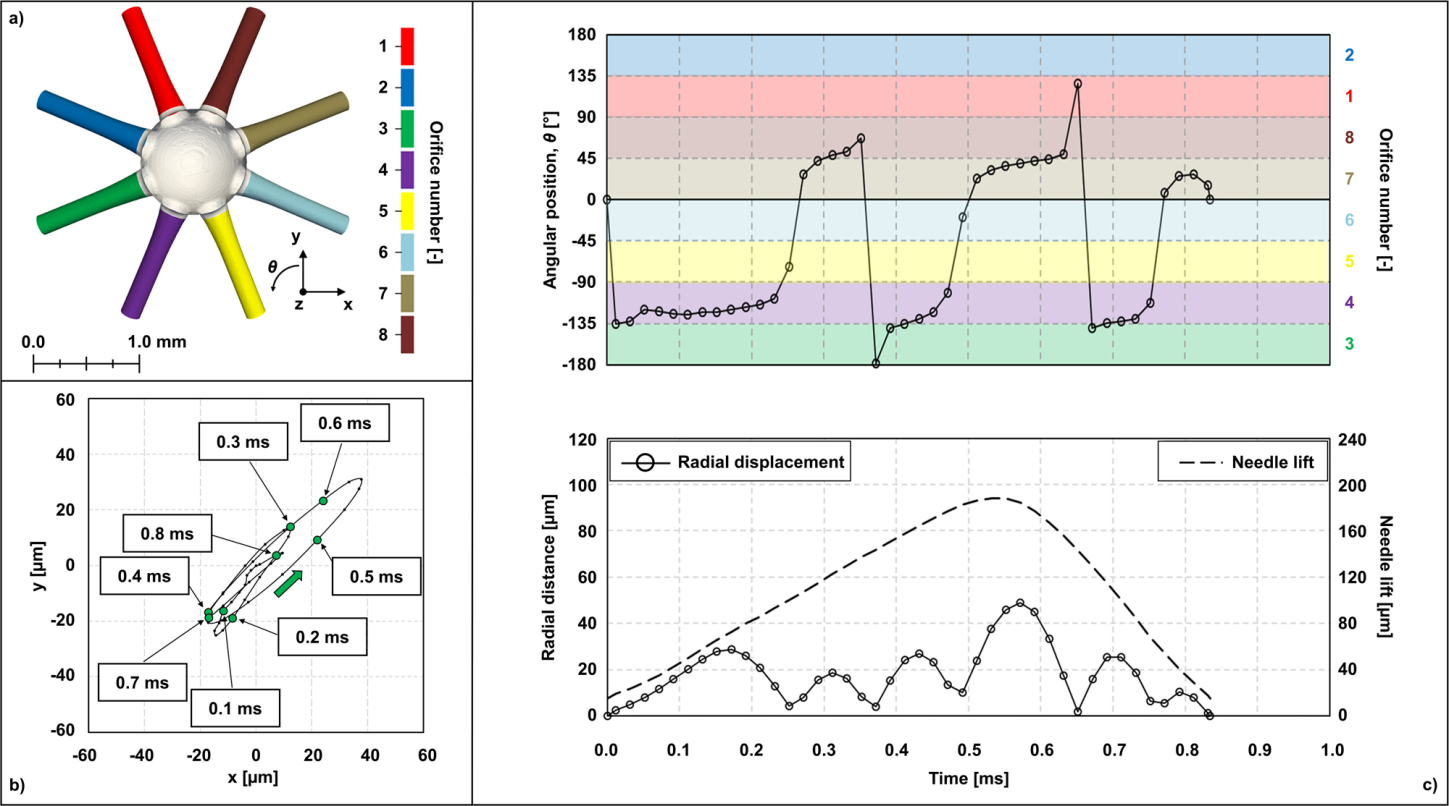
Injector geometry and needle motion paths. **a** reference system and orifice numbering. **b** trajectory of the needle radial motion. **c** decomposition of experimentally measured radial motion trajectory in angular position and radial distance from center of the injector sac, i.e., (*x*, *y*) = (0, 0). The top figure in (**c**) is color-coded according to the orifice to which the needle valve is closer. The bottom figure in (**c**) also reports the experimentally measured needle valve’s lift motion (dashed line with lift values readable on the right axis).

The injection maps used in the current study were extracted from internal flow simulations for which a detailed analysis has been presented in a previous publication by Torelli et al.^6^. The fuel used in the experiments is a straight-run gasoline characterized by a research octane number (RON) of 60. Thus, this fuel exhibits an autoignition reactivity between that of conventional diesel and gasoline, making it a suitable candidate for enabling high efficiency and clean mixing-controlled gasoline compression ignition while harnessing the existing diesel engine architectures. The fuel was pressurized to the desired injection pressure of 100 MPa using a modern common-rail diesel injection system with the injection events triggered by a nominal 0.7-ms energizing current and waveform. A brief description of the methodology used to obtain the injector geometry and the injector needle motion is reported in the Methods section and further information on the experimental setup as well as the detailed injector characterization are reported in the authors’ previous work^7^. Due to the configuration of the experimental setup, the X-ray measurements could only capture one line-of-sight view at a time. Hence, an estimate of the full 3-D needle motion could only be inferred by combining the ensemble averages of two orthogonal views (referred to as *0°* and *90°* views) collected during separate experimental runs and, in principle, uncorrelated. The average axial and radial motion paths employed in this work are reported in Fig. 1b (as a planar trajectory) and **c** (same information as Fig. 1b, but decomposed in angular position and radial distance), together with the reference system and orifice numbering convention (Fig. 1a). The plots in Fig. 1 show that the needle valve moved along a mostly diagonal line aligned with orifices 3–4 and 7–8. The largest oscillation was about 50 µm at 0.57 ms, which occurred at around the same time as when the needle lift was maximum (185 µm at 0.55 ms).

### Analysis of Spray Morphology

**Fig. 2:**
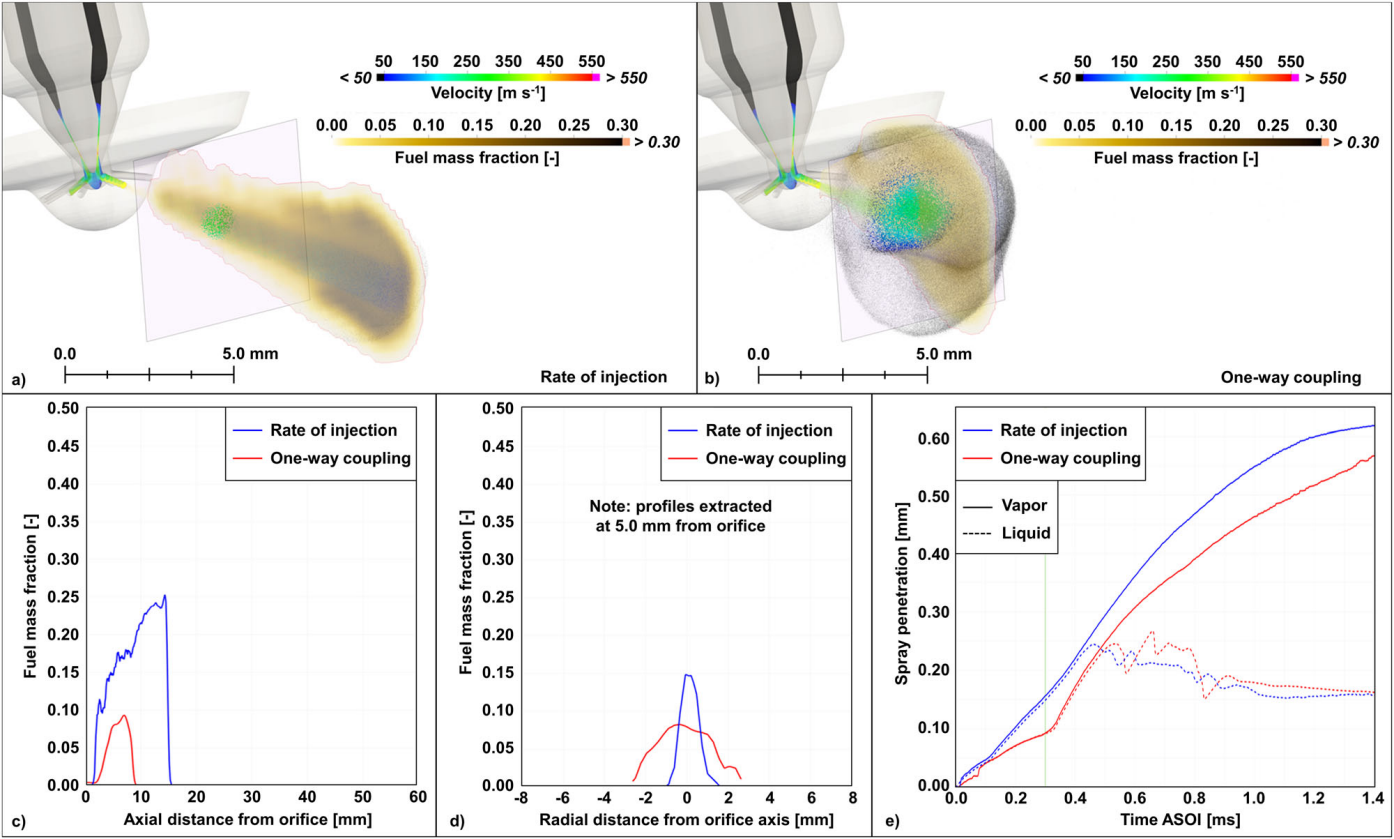
Spray velocity and fuel mass fraction distributions at 0.3 ms aSOI for a simulation run in a constant volume vessel. The fuel mass fraction is plotted on a vertical slice aligned with Orifice 5’s axis**. a** rate of injection (ROI) case. **b** one-way case. The two purple squares are planes used to highlight the spray parcel spatial distribution at 5.0 mm downstream of the injector orifice exit. **c** fuel mass fraction profiles extracted along Orifice 5’s axis. **d** fuel mass fraction profiles extracted at 5.0 mm downstream of Orifice 5 and along a radial coordinate aligned with the vertical direction of the purple squares. **e** liquid and vapor spray penetration vs. time predicted with the two approaches. The green vertical line highlights the *t* = 0.3 ms after start of injection (aSOI) mark.

Figure 2 shows a comparison between the results obtained with the ROI (cf. Figure 2a) and the one-way coupling (cf. Figure 2b) approaches. Here the focus is on one orifice only (i.e., Orifice 5) to highlight the differences in spray morphology between the two approaches. With ROI, the user assigns a cone angle, i.e., the solid cone within which the Lagrangian parcels are introduced and distributed at each timestep. While the user can impose time-varying cone angle profiles that rely on experimental observations or the use of empirical models^8^, this parameter is typically assigned as a constant value. In this case, the spray will evolve in a way that is self-consistent with respect to the elapsed time, across the injection event. In addition, as this parameter is assumed to be the same for all orifices, each spray plume will evolve identically, therefore not revealing any orifice-to-orifice variability. With the one-way coupling approach, on the other hand, the cone-angle is not assigned a priori. Instead, it is the result of the instantaneous velocity vector orientation computed at every cell located on the orifice exit plane, during the internal flow simulations. This allows the ensuing spray to follow the temporal evolution of the internal flow and exhibit transient behavior that can last for relatively short times compared to the duration of the entire injection event. It is clear from Fig. 2a, b that at around 0.30 ms after the start of the injection (aSOI), the internal flow dynamics result in a widening of the spray cone which leads to a temporarily hollow cone, as well as considerable differences in fuel vapor mass fraction distribution (cf. Figure 2c, d), and liquid and vapor penetrations (cf. Figure 2e). Similar behaviors and differences between the two approaches were observed across the entire injection event and for all orifices.

**Fig. 3:**
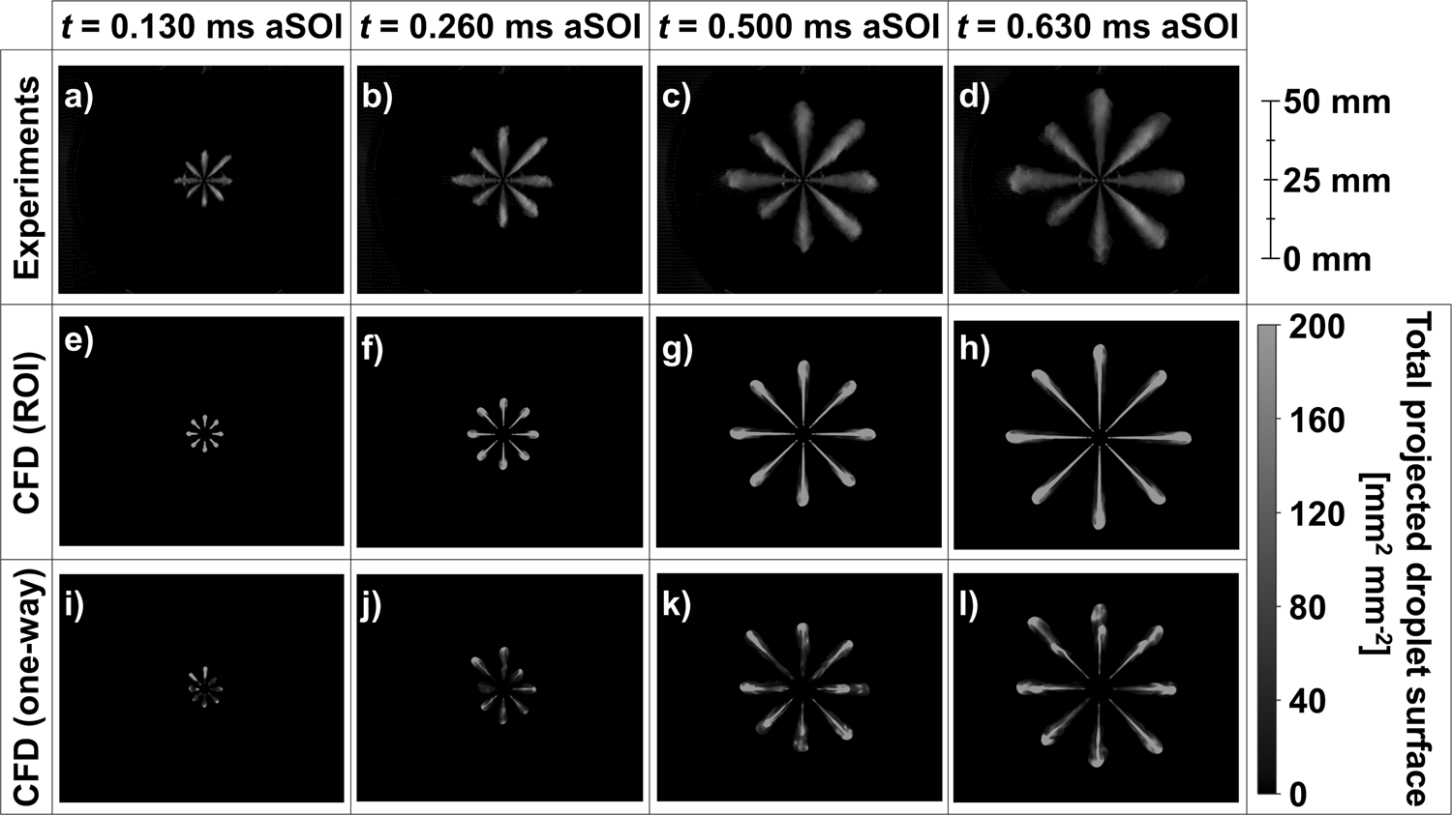
Temporal evolution of spray morphology. **a**-**d** Mie scattering experiments carried out with the medium-duty (MD) injector. **e**-**h** total projected droplet surface field extracted from Lagrangian-Eulerian (L-E) simulations performed using the rate of injection (ROI) approach and the heavy-duty (HD) injector. **i**-**l** total projected droplet surface field extracted from L-E simulations performed using the injection profiles maps obtained from the internal-nozzle-flow simulations of the HD injector. Note: the computational fluid dynamics (CFD) results at *t* = 0.130 ms after start of injection (aSOI) use different color bar limits (0–20 mm^2^ mm^−2^) for improved visibility of the spray morphology.

Unfortunately, no optical experimental data about the spray evolution were available for this particular HD injector unit. On the other hand, Mie scattering optical spray measurements of a slightly smaller, but very similar, eight-hole diesel injector used for medium-duty (MD) applications (produced by the same injector manufacturer) were available with the same straight-run gasoline fuel. This dataset provided qualitative validation of the behavior observed in the one-way coupling spray simulations, which was not witnessed with the ROI approach. Figure 3 reports the temporal evolution of the multi-plume spray as observed in the experiments with the MD injector (cf. Figure 3a–d), as well as the spray morphology predicted by the ROI (cf. Figure 3e–h) and one-way coupling (cf. Figure 3i–l) approaches using the HD injector. To ensure qualitative consistency between the experimental Mie scattering imaging and the CFD results, the simulated liquid spray was processed to obtain a planar field of total projected droplet surface. This was achieved by line-of-sight integration of the total droplet surface field of the Lagrangian parcels onto a uniform grid of 0.1 mm size. Even though the comparison is made with different injectors, it can be seen that spray morphology and the orifice-to-orifices differences predicted with the one-way coupling approach are qualitatively very similar to those observed in the experiments. On the other hand, the ROI approach showed a very consistent behavior among all orifices as clearly seen from the temporal evolution of the spray plumes shown in Fig. 3e–h. The lack of plume-to-plume variability with the ROI approach is a clear indication of the limited ability of this model to replicate the hole-to-hole differences in time and space, which are otherwise observed when the one-way coupling approach is employed. One final note must be made about the CFD images shown in Fig. 3 for the *t* = 0.130 ms aSOI time stamp. At that time, the overall peak values of the total projected droplet surface were much smaller than those that were found at later timesteps, due to more limited breakup during the early stages of the injection. This required the use of a smaller upper limit of 20 mm^2^ mm^−2^ to enhance the visibility of the spray and ensure a satisfactory visual comparison against the Mie scattering experiments.

### Effect of spray variability in engine simulations

**Fig. 4:**
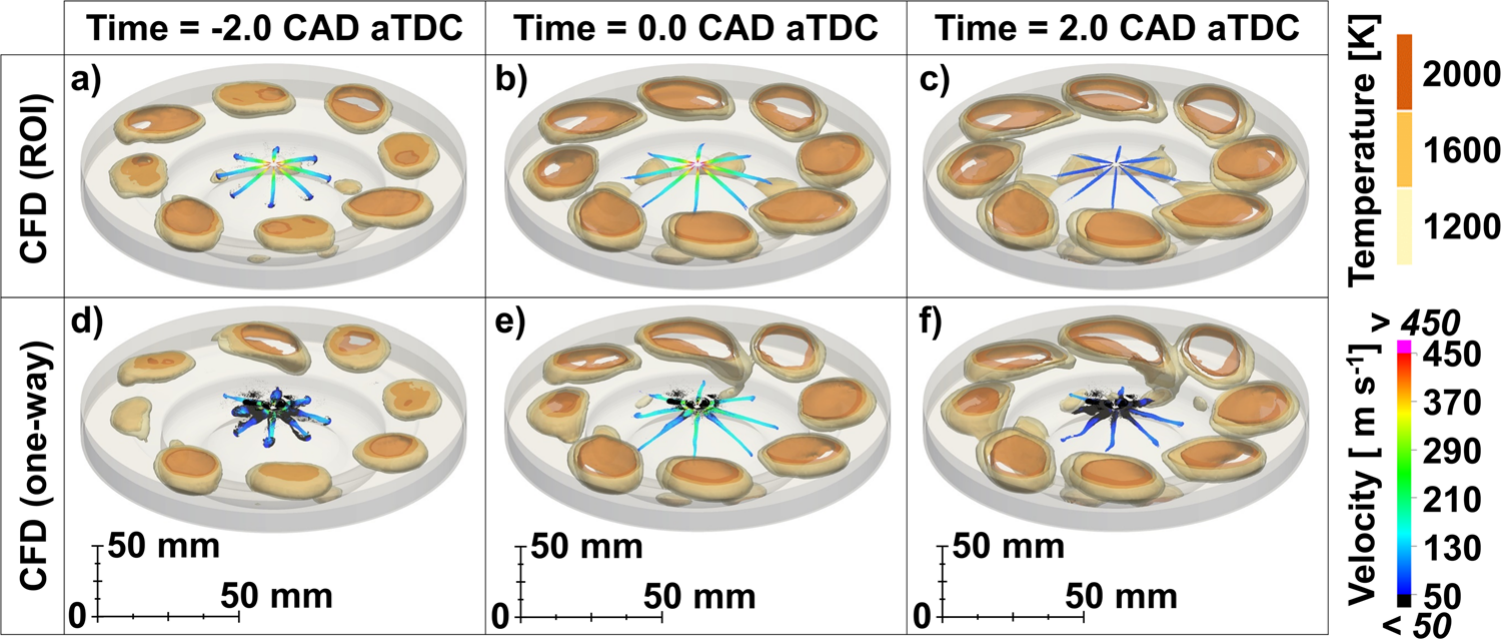
Flame propagation near top dead center (TDC) in a compression ignition engine simulation between *t* = −2.0 and *t* = 2.0 crank angle degrees (CAD) after TDC (aTDC). Iso**-**surfaces of temperature identify the flame front, while the fuel spray parcels are colored by their velocity. **a**-**c** Computational fluid dynamics (CFD) predictions obtained using the rate of injection (ROI) approach. **d**-**f** CFD predictions obtained using the one-way coupling approach. With one-way coupling, due to the transient widening of the spray, many parcels are injected to the sides where drag effects become relatively more important and cause the droplet to slow down considerably (cf. black parcels). These parcels are also more likely to impinge on the cylinder head creating a fuel film that persists during the early stages of the combustion, as visible from the visualization in subfigure (**f**).

The two time-series of images in Fig. 4 show engine combustion results obtained with the ROI (cf. Figure 4a–c) and the one-way coupling (cf. Figure 4d–f) approaches. The fuel mass flow rate profile used for the ROI case was extracted from the same internal nozzle flow simulation that provided the injection maps for the one-way coupling case. This strategy allowed for formal consistency between the two approaches in terms of total injected mass and mass flow rate. The modeling setup of the engine cases and the engine specifications are described in greater detail in the Methods section. It can be seen that the eight spray plumes obtained with the ROI approach are all similar to each other and do not show any visible plume-to-plume differences. With the use of one-way coupling, consistent with what has been shown in Fig. 3, each plume has undergone a different temporal evolution which has influenced the evaporation and mixing of the fuel, in turn creating mixture inhomogeneity and slightly different flame structures (shown by the iso-surfaces of the gas temperature field) which also evolved with different burning rates in time.

**Fig. 5:**
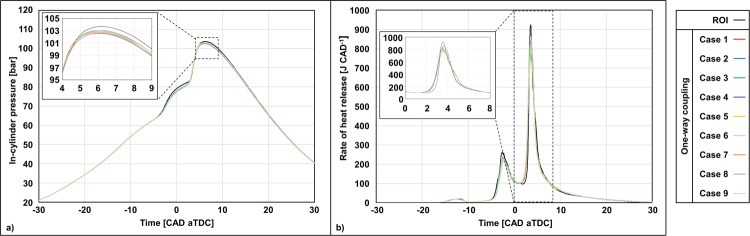
Thermodynamic performance of the engine using the rate of injection (ROI) and one-way coupling approaches. **a** In-cylinder pressure vs. time. **b** Rate of heat release vs. time. For both quantities, a zoomed version of the plot is provided to show the differences in peak at crank angle degrees (CAD) values immediately after top dead center (aTDC).

Figure 5a, b show the in-cylinder pressure and the rate of heat release (RoHR) vs. time during the injection and combustion phases of the engine cycle for a series of cases. For the one-way coupling approach, eight additional engine simulations characterized by eight different injection maps were performed, bringing the total number of one-way coupling engine cases to nine. In addition to the average needle motion map used for the simulation shown in Fig. 4 (whose needle motion profiles are depicted in Fig. 1), eight additional internal flow simulations were performed to create as many new injection maps. The only difference among the overall nine cases was in the use of different needle motion profiles characterized by a unique radial motion displacement profile for each case. To ensure consistency with the experiments, the radial motion profiles were randomly sampled from the *0°* and *90°* view experimental datasets and combined to obtain new needle motion trajectories compatible with the variability observed in the experiments.

The analysis of the results in Fig. 5b shows that the low-temperature RoHR, occurring between −20 and −10 crank angle degrees (CAD) after top dead center (aTDC), is practically identical for all cases. On the other hand, most of the differences, although minor, are observed in two distinct periods with the first one being between −5 CAD aTDC and TDC and the second one between 2 and 6 CAD aTDC. During the earlier period, the ROI case shows a slightly higher RoHR peak (cf. Figure 5b), indicating the occurrence of a slightly faster combustion of the fuel-air mixture. This is reflected in the pressure trace as well, which shows a higher and slightly more rapid pressure increase between −3 and 3 CAD aTDC (cf. Figure 5a). A similar behavior is also observed in the peak pressure during the second period (i.e., between 2 and 6 CAD aTDC). Here, the ROI case reaches a peak pressure of 103.4 bar at 6.20 CAD aTDC. Every one-way coupling case shows a lower peak pressure which ranges between 102.5 bar (Case 7) and 103.1 bar (Case 6). Very small differences are also observed for the time at which the peak pressure is found, which generally occurs earlier than what was observed for the ROI case, ranging between 6.01 CAD aTDC (Case 9) and 6.14 CAD aTDC (Case 4). Another difference between the ROI case and the one-way coupling cases can be observed in the zoomed plot of Fig. 5b. Here, the RoHR trace of the ROI case shows a delayed rise compared to all one-way coupling cases.

**Fig. 6:**
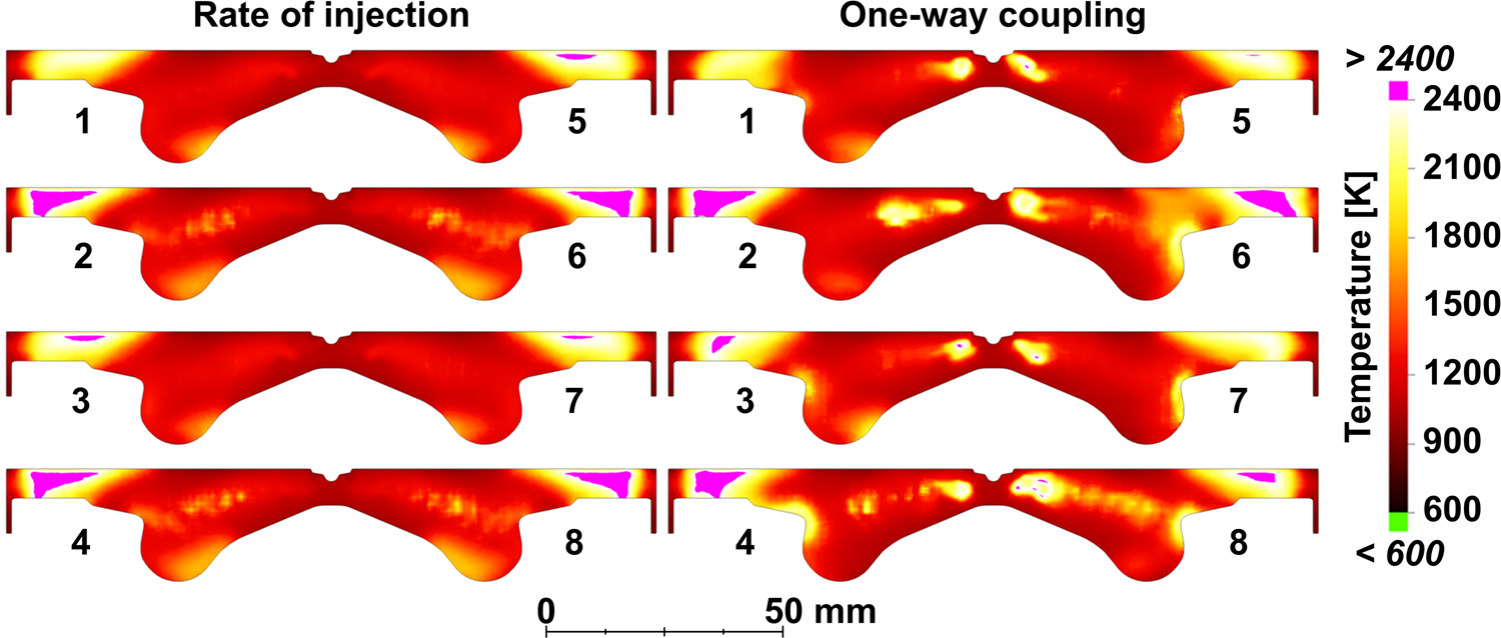
Gas temperature distributions at *t* = 5.0 crank angle degrees (CAD) after top dead center (aTDC) using the rate of injection and the one-way coupling (Case 1) approaches. The temperature is plotted on vertical slices that are aligned with the orifice axes. The orifice numbers are marked following the convention used in Fig. 1a.

As noted earlier, the only difference between the ROI and the one-way coupling cases is in the methodology used to initialize the fuel spray in the computational domain. Hence, it can be assumed that all the differences discussed above are likely due to the interaction between the spray and the gaseous flowfield. This speculation is in line with the discussion of Fig. 4 and is further confirmed in Fig. 6 where a direct comparison of the gas temperature distributions between the ROI and one of the one-way coupling cases (i.e., Case 1) is presented.

With the ROI approach, the gas temperature distributions are visibly similar for all orifices as a result of comparable interactions between the spray plumes and the surrounding gas phase. In contrast, the temperature plots of the one-way coupling case show larger differences from orifice to orifice with high-temperature pockets developing in the near-nozzle regions that are not observed with the ROI approach. These pockets are due to a localized, enhanced fuel evaporation caused by the initial spreading of the spray cone angle, as also shown in Fig. 2b–d. In addition, due to the different development of each spray plume (cf. Figure 3 and Fig. 4) and their interaction with the surrounding gas, the value and locations of the temperature peaks are visibly different from orifice to orifice. It is clear that these orifice-to-orifice differences introduced by the use of the one-way coupling approach have important consequences on the combustion behavior. It is expected that the mixture’s temperature and composition inhomogeneities will impact the paths followed by the chains of chemical reactions, eventually affecting the emission signature.

**Fig. 7:**
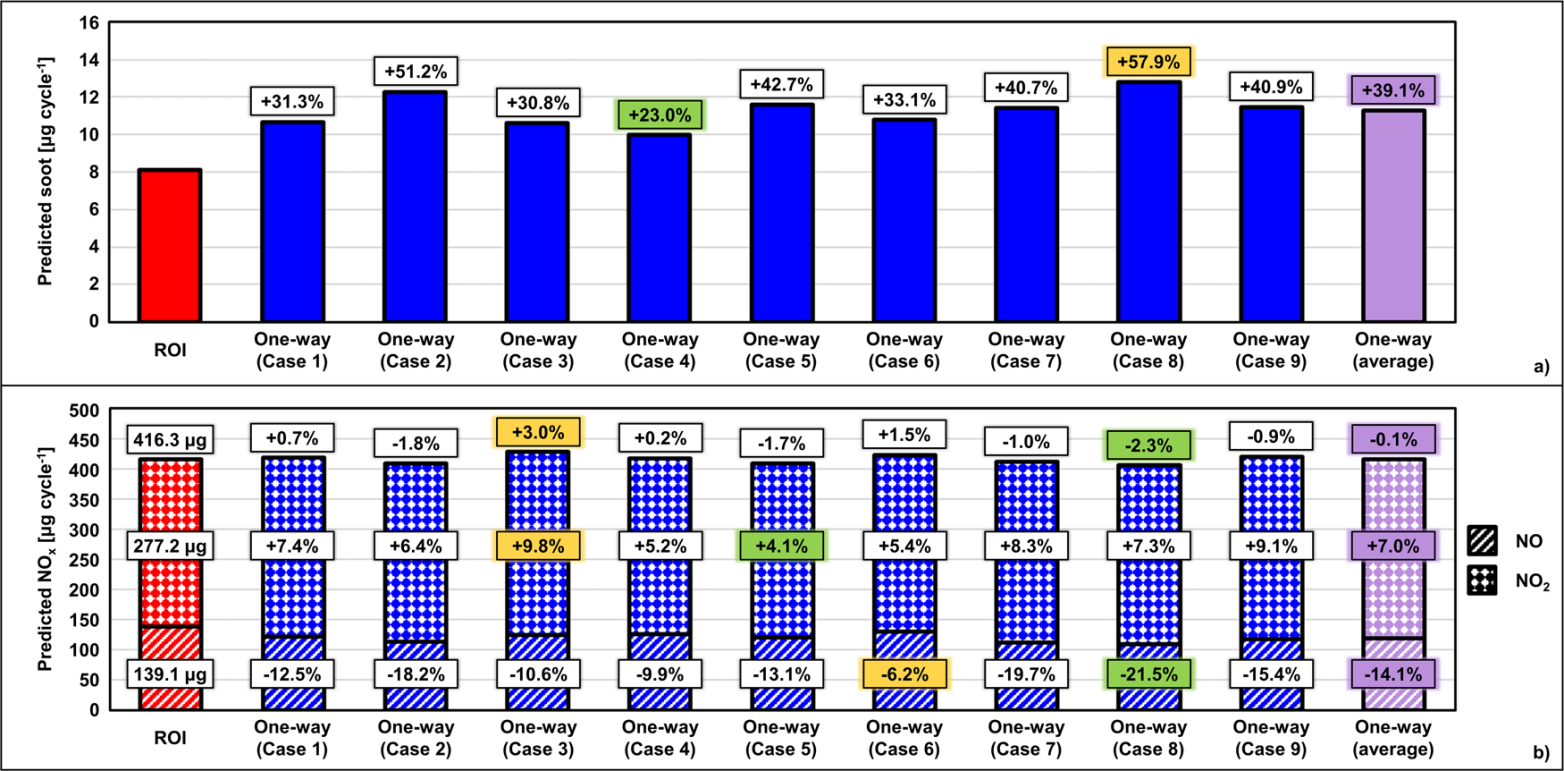
Comparison of single-cycle emission predictions using the rate of injection (ROI) and one-way coupling approaches. **a** Soot. **b** Nitrogen oxides (sum of NO and NO_2_). In all plots, the red bars are used for the ROI results, the blue bars for the nine one-way coupling cases, and the purple bars for the average of the one-way cases. The green and yellow boxes are respectively used to highlight the lowest and highest value of predicted emissions for the one-way cases. The percentage values in the boxes represent the differences between the one-way predictions and the ROI case (used as reference).

To highlight the consequences of the differences discussed above, Fig. 7a shows a comparison of the predicted soot between the two injection models. The soot predictions show that, not only the average predicted soot was 39% higher for the one-way coupling cases compared to ROI, but also that the use of different injection maps resulted in considerable variability of the predicted soot that ranged between 23% and 58% more than the ROI case. Similar conclusions can be made by evaluating the NO_x_ emission signature (cf. Figure 7b). While the total NO_x_ did not differ substantially between the ROI and the one-way coupling approach, their composition showed considerable differences in terms of NO and NO_2_, with important implications on the functionality of the aftertreatment systems used to abate such emissions at the tailpipe level. This analysis highlights the ability of this approach to not only capture orifice-to-orifice variability and its effect on combustion behavior, but also shot-to-shot injection differences and their ultimate impact on emissions, which would be otherwise missed if a typical ROI approach were to be used.

## Discussion

This work demonstrates the feasibility of an end-to-end approach that links the internal flow of automotive injectors to the ensuing fuel spray formation and subsequent fuel mixing, combustion, and emissions signature in internal combustion engine applications. By comparing the industry-standard ROI approach and a static-coupling methodology known as one-way coupling, this study shows that retaining as much information as possible from the internal flow has important implications on the behavior of the ensuing spray. In particular, it has been shown that the plume-to-plume variability observed in optical spray experiments could be replicated, at the very least qualitatively, by initializing the Lagrangian spray via spatiotemporally resolved maps extracted at the injector’s orifice exits and stored at runtime. The orifice-to-orifice variability observed inside the injector via internal flow simulations resulted in transient spray behaviors that could only be predicted using the one-way coupling methodology. In particular, fuel vapor distribution as well as liquid and vapor penetration vs. time were strongly affected by the timing and occurrence of transient widenings of the spray cone angle and the temporary formation of hollow cone spray structures. These spray features influenced the fuel-air mixing in the engine and the combustion behavior, which in turn, resulted in sensibly different emission signatures in terms of soot and nitrogen oxides. Hence, the ability to provide a more accurate, spatiotemporally varying initialization of the Lagrangian parcels in terms of velocity, size, and mass has the potential to lead to improved drag estimates and in turn, a more accurate liquid-gas momentum and energy coupling. This allows all spray submodels to operate with improved boundary conditions from both the liquid and the gas sides, which will result in more accurate submodel outcome predictions. Therefore, as some spray submodels keep improving, such as is the case of the one-way coupling injection model used in this work, other submodels must improve too in order to maximize the benefit of using more accurate approaches. Following, the effect of shot-to-shot variability was also explored by applying different injection maps resulting from internal flow simulations characterized by different needle valve motion profiles. Nine needle motion profiles were sampled within the range observed experimentally, allowing for an estimate of the effect that shot-to-shot differences in the internal nozzle flow have on the single-cycle emission signature. It is shown that, not only the average predicted soot was larger than that predicted with the ROI approach, but also that the associated cycle-based soot emissions can vary considerably from one cycle to another. This aspect can become very important if injection-induced variability should result in different distributions of soot particle number and size over different cycles. Accounting for shot-to-shot injection variability would allow to achieve a more accurate characterization of the soot particle population than what can be obtained with the ROI approach. On the other hand, this type of analysis highlights a clear need for more accurate phenomenological or detailed soot models that are capable of particle number and size predictions, as opposed to the model used in this work, which can only provide mass-based predictions. Similar conclusions could be drawn for the nitrogen oxide emission signature. Although the total NO_x_ did not differ substantially between the ROI case and the average of the one-way coupling cases, its composition (i.e., NO + NO_2_) was considerably different. This is a clear indication that the combustion of the fuel-air mixture followed different chemical paths as a result of the shot-to-shot and plume-to-plume differences in mixing behavior resulting from the use of the one-way coupling approach. It should be noted that this study focused on only one operating point and one specific engine-out NO_x_ level. A more comprehensive exploration of operating conditions would allow for a better-informed understanding of the engine behavior and potentially lead to improved guidelines for optimum engine design. Nevertheless, this work highlights the importance and the effect that physics-based, informed injection conditions have on the mixing and combustion behavior of internal combustion engines. This is especially impactful when designing a low-NO_x_ combustion engine able to comply with future ultra-low heavy-duty tailpipe NO_x_ standard of 0.02 g hp-hr^−1^ while ensuring a viable NO_x_-soot tradeoff.

## Methods

The work presented in this manuscript relies on two different kinds of simulations: (1) an Eulerian-Eulerian mixture modeling approach for the solution of the injector’s internal flow and (2) a Lagrangian-Eulerian (L-E) representation of the ensuing spray.

### CFD modeling framework and setup: internal flow Eulerian-Eulerian simulations

The computational setup is consistent with previous work from the authors^6,7,9,10^. All simulations were performed with the CONVERGE CFD code^11^ using an unsteady Reynolds-averaged Navier Stokes (RANS) formulation closed by the standard *k-ε* turbulence model^12^. For the internal flow calculations, the individual species solution method, also known as homogenous mixture model, is utilized to describe the multiphase nature of the flowfield. With this method, a density-based solver is used to transport total mass, momentum, energy, and species. Once the transported species are known in each computational cell, the code calculates the void fraction field. This implies that the void fraction is not transported directly, instead each individual gas species is solved with dedicated transport equations. The total gas mass in each cell is then determined as the sum of each gas species mass. The difference between the total cell mass and the gas mass leads to the knowledge of the liquid mass, allowing for the determination of the local void fraction. All equations are solved with second-order space and first-order time accuracy, and with a Courant–Friedrichs–Lewy condition that, depending on the minimum mesh size, results in maximum timesteps in the order of 10 ns for the Eulerian-Eulerian internal flow simulations.

The mesh structure generated by the CONVERGE software relies on a cut-cell approach, i.e., the computational domain is defined as the intersection between a closed 3-D surface representing the fluid region of interest and a Cartesian grid whose base mesh size is set as 160 µm for all the internal flow simulations reported in this work. Embedded mesh refinements are applied to regions of the domain where finer mesh resolution is deemed necessary. Specifically, minimum sizes of 20, 10, and 5 µm are respectively used in the injector sac, inside and immediately downstream of the orifices, and in correspondence of the needle seat. The law-of-the-wall model^13^ is employed to determine the velocity in every near-wall first cell and the mesh sizes reported above ensured that the local *y*^*+*^ value is within the range of validity of the wall functions. The injector geometry is a realistic representation of a modern, heavy-duty, eight-hole, diesel injector. The 3-D surface file is obtained as the combination of X-ray-informed portions of the geometry which were reconstructed using a computer-aided-drawing software (e.g., the region upstream of the needle tip) with other portions (i.e., the needle tip, the injector sac, and the eight orifices) that were obtained using computed tomography applied to high-resolution (1.17 µm pixel^−1^) X-ray scans performed at the 7-BM beamline at the Advanced Photon Source (APS) at Argonne National Laboratory^14^. A detailed description of the experimental techniques as well as the characterization of the injector are reported in the authors’ previous work^7^.

As the solver is designed to handle moving boundaries, transient 3-D motion profiles are applied to describe the movement of the injector needle valve. These motion profiles are also based on X-ray experimental measurements conducted at Argonne’s APS^7^, beamline 32-ID^15^. These 3-D profiles account not only for the needle lift, i.e., the motion describing the opening and closing of the needle valve, but also for the radial off-axis motion due to mechanical vibrations of the needle occurring during the injection event. More details on the needle motion profiles have been published in past studies^7^.

All fluid phases, i.e., liquid, vapor, and non-condensable gases, are modeled as compressible. A barotropic-fluid assumption is used to account for the compressibility of the liquid phase and its mathematical formulation is as follows:

1 $${\rho }_{{{\mbox{l}}}}={\rho }_{{{\mbox{l,ref}}}}{{{\mbox{e}}}}^{\left(\frac{{P}_{{{\mbox{l}}}}-{P}_{{{\mbox{l,ref}}}}}{{B}_{{{\mbox{l}}}}}\right)}$$

In Eq. (1), *ρ*_l_ is the density of the liquid fuel at the local liquid pressure, *P*_l_, while *ρ*_l,ref_ and *P*_l,ref_ are density and pressure of the liquid phase at the reference ambient conditions (i.e., *P*_l,ref_ = 1.013 bar and reference temperature, *T*_l,ref_ = 358 K). Finally, *B*_l_ is the bulk modulus of the liquid phase.

The thermodynamic behavior of fuel vapor and non-condensable gases is described using the Redlich-Kwong cubic equation of state^16^. The non-condensable gases present in the liquid fuel are modeled by imposing a trace mass-fraction amount of molecular nitrogen, N_2_, at the domain inlet equal to 2.0×10^−5^. This value is estimated on the bases of considerations of gas phase solubility provided by Battistoni et al.^17^. The physical properties of the straight-run gasoline fuel used in this study are defined as functions of the liquid temperature and derived by means of Aspen HYSYS^18^, as documented in previous work by the authors^7,9^. The phase change due to the occurrence of in-nozzle cavitation is modeled via the homogeneous relaxation model proposed by Bilicki and Kestin^19^. This model accounts for the phase change occurring under non-equilibrium thermodynamic conditions in which the liquid phase is exposed to local pressures lower than its saturation pressure.

### CFD modeling framework and setup: static-coupled Lagrangian-Eulerian spray simulations

A Lagrangian-Eulerian framework is used to describe the mutual interaction between the liquid spray originating at the injector’ orifice exits and the surrounding gas phase. The fuel spray is modeled using Dukowicz’s discrete droplet modeling approach, whose mathematical formulation allows to avoid numerical diffusion and is known to be computationally affordable^5^. This approach establishes a two-way gas-liquid momentum coupling between the gas phase (treated in an Eulerian fashion) and a cloud of Lagrangian parcels that models the distribution of the liquid phase inside the computational domain. The parcels are statistical entities used to describe the likelihood that a certain mass of liquid is located at a certain location and is characterized by a series of morphological (e.g., droplet diameter, number of droplets, droplet velocity, etc.) and thermophysical (e.g., temperature, density, viscosity, surface tension, etc.) properties. Furthermore, the parcels are also able to exchange mass (from liquid to gas) and energy (two-way) owing to the mutual interaction between the liquid and gas phases. The validation of this approach with the CONVERGE CFD code^11^ has been extensively reported in the previous publications^20–23^ and will not be discussed here. Second-order spatial discretization is used to resolve the flowfield, while time-dependent quantities are modeled with first-order accuracy, using a Courant-Friedrichs-Lewy-based time-step limited to a maximum of 5.0 × 10^−^^6 ^s. Consistent with the internal flow simulation approach, an unsteady RANS formulation is chosen to model the turbulence closure by means of the re-normalization group RNG *k-ε* model^24^. The choice of such methodology creates opportunities for developing models that can support very large numbers of simulations while retaining reasonable computational time frames, such as those required by simulation campaigns aimed at optimizing designs for ICE systems^25,26^. The primary liquid atomization of the spray is achieved by means of the blob injection model^27^, while the secondary breakup is modeled using the Kelvin Helmholtz-Rayleigh Taylor KH-RT model in the version proposed by Patterson and Reitz^28^. Following a recent spray validation study by Torelli et al.^29^, the O’Rourke’s numerical scheme is used to account for droplet collisions and coalescence^30^. The Frossling correlation is employed to account for droplet evaporation (i.e., liquid-to-gas phase change) assuming a uniform droplet temperature during the thermal transfer between the droplet and the surrounding gaseous environment^31^. The quantification of the coupled momentum exchange between the gas and liquid phases is estimated using the dynamic drag model^32^. The introduction of the spray parcels in the computational domain is achieved by means of a one-way coupling injection model, which is described in greater detail in the following subsection.

The combustion is modeled using a previously validated setup proposed by Pei et al.^25^, where the SAGE detailed chemistry combustion model^33^ is combined with a multi-zone approach^34^. The fuel’s physical properties for the liquid phase are the same as those used for modeling the liquid in the internal flow simulations. The chemistry of the fuel is modeled using a primary reference fuel mechanism from Liu et al.^35^. The Hiroyasu model^36^, coupled with the Nagle and Strickland-Constable model^37^ is used to estimate the soot oxidation, while NO_x_ predictions are carried out by means of a four-species, 13-reaction reduced mechanism^38^. The base mesh size in the domain is set to 1.4 mm, while adaptive mesh refinement based on velocity and temperature gradients is used to reach a minimum mesh size of 0.35 mm in those regions where the gradient-based refinement criteria are met. Finally, fixed-embedded grid refinements of 0.35 mm in the near-nozzle regions guarantee an adequate mesh resolution during the early transients of the injection. More information about the engine specifications can be found later in this manuscript, while a detailed description of the mesh and combustion setup as well as its validation against experimental results can be found in the previous study by Pei et al.^25^.

### The One-Way Coupling Injection Model

The modeling of liquid sprays in the Eulerian-Lagrangian framework typically relies on what is commonly referred to as the rate of injection (ROI) approach. This methodology requires the a-priori knowledge of inputs such as fuel temperature, injection rate profile, amount of injected mass, injector discharge coefficient, *C*_d_, and spray cone angle. These inputs are generally obtained via experimental characterization of the injection system, a procedure that requires dedicated facilities and trained personnel. An alternative to experimentally measured inputs is offered by one-dimensional dynamic system models that are able to return the above-mentioned inputs on the basis of considerations about the typology and nominal geometry of the nozzle, injector needle energizing current vs. time profiles, operating conditions (i.e., injection pressure, back pressure, fuel and ambient temperatures), and fuel properties^39,40^. However, the ROI approach and the one-dimensional models generally carry little to no information about shot-to-shot variability as well as hole-to-hole differences in multi-hole injectors. Such variabilities tend to arise during the transient stages of the injection (e.g., needle valve opening and closing events)^41^ and numerical studies have indicated the possibility that hole-to-hole differences are linked to off-axis radial displacement of the needle^6,7,42^ that occur for either mechanical or fluid-dynamic reasons^43,44^.

By means of static coupling methodologies such as the one-way coupling approach used in this study, it is possible to retain a detailed knowledge of the internal flow’s spatiotemporal evolution at the orifice exits and utilize such information to initialize a Lagrangian spray. From the perspective of its interaction with the surrounding Eulerian gas phase, the spray is formally identical to a spray initialized with the ROI approach. The main differences between the two approaches lie in the set of inputs needed to introduce the computational parcels in the CFD domain and, most importantly, in the ability of this approach to retain detailed transient spatiotemporal features resulting from the evolution of the injector’s internal flow.

Several authors have reported on static-coupling approaches that aim to retain the detailed information obtained from the internal flow and pass it down to the Lagrangian spray. Some relevant examples can be found in the works of von Berg et al.^45^, Masuda et al.^46^, and Battistoni et al.^47^ von Berg et al.^45^ proposed a methodology based on the analysis of the cavitation observed inside automotive injectors and linked the cavitating flow information with a static-coupling, advanced primary breakup model which is applied on a subgrid located inside the injector orifice. In their model the Lagrangian parcels are released from a liquid core region and their characterization is determined on the basis of turbulent kinetic energy and turbulent energy dissipation in the nozzle orifice. This approach allowed to predict asymmetries in the cavitation structures arising within a simplified nozzle geometry, which were reflected on the spray shape as well as on the inhomogeneous distribution of droplet properties within the spray cone. The work by Masuda et al.^46^ continued on a similar path as von Berg et al.’s and applied the model described above^45^ to a valve-covered orifice injector using a realistic one-dimensional needle lift motion profile. Masuda et al. were not only able to show similar flow and spray features to those observed in the work of von Berg et al., but also the effect of transient needle motion on the widening and narrowing of the near-nozzle cone angle, also referred to as the microscopic cone angle by the same authors. However, Masuda et al. showed that, for the specific injector used in their study, the effect of the microscopic cone angle did not propagate to the overall macroscopic spray cone-angle, as opposed to what has been observed in this current work and also shown in the experimental work of Jung et al.^48^. Finally, Battistoni et al.^47^. used a similar approach to that of von Berg et al. and Masuda et al. to investigate the behavior of biodiesel and diesel fuels and the ability of the static-coupling, primary breakup model mentioned above to capture the effect of different fuel physical properties.

Other researchers have explored higher-accuracy approaches such as the Eulerian-Lagrangian Spray Atomization ELSA model first introduced by Vallet et al.^49^, further developed by Blokkeel et al.^50^, and recently adopted by Anez et al.^51^ in which the ELSA model was coupled with interface capturing methods for diesel injector applications. On a similar note, some research groups have proposed direct transition of isolated drop-like structures from the Eulerian domain to a Lagrangian representation^52–54^. These methodologies^49–54^ are all characterized by high accuracy of the near-nozzle solution. However, this benefit comes at the cost of requiring particularly small mesh sizes (*O*(μm)) and time steps (*O*(ns)) in the near-nozzle region in order to achieve an accurate solution of the smaller liquid structures before the transition from the Eulerian to the Lagrangian domain can occur. This requirement poses an often-insurmountable limitation to the application of these methods to engineering-level combustion engine simulations.

**Fig. 8:**
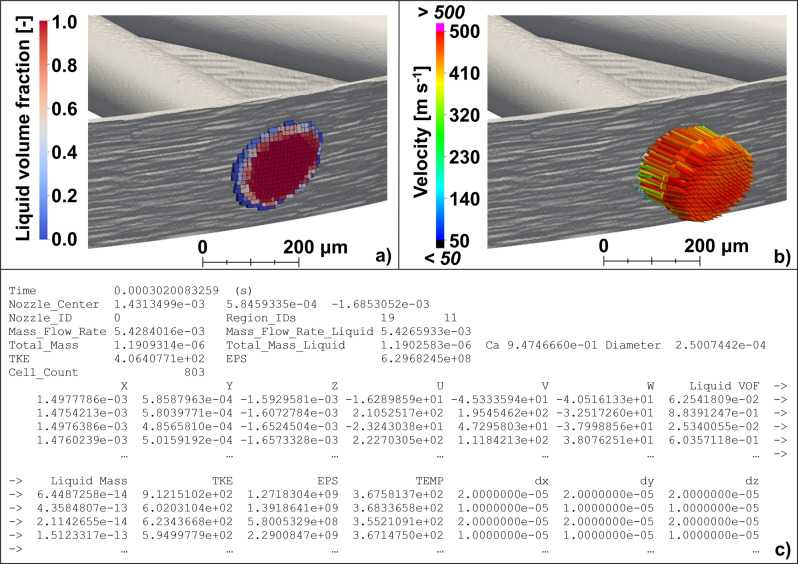
Details about the information stored in the one-way coupling maps. **a** Example of liquid volume fraction distribution. **b** Example of velocity vectors. **c** An excerpt of the one-way coupling map file.

In the work reported in this manuscript, the one-way coupling injection model’s only requirement is the existence of a spatiotemporally resolved map that contains a series of quantities extracted at runtime from the internal flow simulations, in correspondence of the orifice exists. In the implementation of the one-way coupling model available in CONVERGE and first proposed by Quan et al.^55^, said quantities are: spatial location of the computational cells where the quantities have been computed, velocity vector components, local liquid volume fraction (LVF) and mass, turbulent kinetic energy and its dissipation rate (needed for unsteady RANS-based calculations only), temperature, and dimensions of the sampled cell along the X, Y, and Z directions. Such maps are created with a user-specified temporal frequency and for each orifice of the injector. Other necessary information is also stored at each time instance in which the maps are generated. Some examples are the orifice outlet center coordinates, the instantaneous mass flow rate, the area contraction coefficient, *C*_*a*_, etc. Figure 8a, b shows two visual examples of the quantities that are stored in the one-way coupling maps, namely LVF and velocity vectors, while Fig. 8c shows an excerpt of a one-way coupling map file.

The spray liquid parcels are introduced at every location reported in the map file (i.e., X, Y, and Z columns of Fig. 8c) if their local LVF is larger than a user-specified threshold. In the current work, the LVF threshold is set to 0.4, which is the recommended value reported by the CONVERGE manual based on model calibration for diesel-like injectors. This implies that the liquid parcels are introduced at each given location only if the local, instantaneous LVF is larger than 40% of the total cell volume. This is done to ensure that the effect of super-cavitation (i.e., fuel vapor generated inside the orifice via cavitation, and crossing the orifice outlets) on the liquid distribution of the spray is properly captured. It should be noted that this choice has a limited effect on the local injected mass as the threshold is volume-based and therefore the mass fluxes originating from those cells with LVF < 0.4 carry only a small amount of liquid fuel mass. Nevertheless, to ensure mass conservation, all the liquid originating from the excluded cells is uniformly spread across the cells that satisfy the LVF condition. A random perturbation is then applied to each parcel locations within a volume of *dx*, *dy*, *dz* dimensions (cf. last three columns of Fig. 8c). At these locations, the one-way coupling algorithm introduces one or more parcels at each computational time-step according to a user-specified minimum parcel mass and the total liquid mass flowing through the cell between the current time-step and the next one. All the liquid physical properties (e.g., density, viscosity, surface tension, heat of vaporization, etc.) are assigned to every parcel as a function of the local temperature stored in the map file. Each single parcel represents a certain number of droplets whose diameter, *d*, is calculated as a function of the hydraulic diameter of the orifice outlet, *d*_orif_, and the instantaneous *C*_a_:

2 $$d=\sqrt{{C}_{{{\mbox{a}}}}}\,{d}_{{{\mbox{orif}}}}$$

In which *C*_a_ is defined as the ratio between the effective and the geometric orifice areas and is calculated considering the effective area crossed by the liquid phase:

3 $${C}_{{{\mbox{a}}}}=\frac{{A}_{{{\mbox{eff}}}}}{{A}_{{{\mbox{orif}}}}}=\frac{\sum ({{{\mbox{LVF}}}}_{{{\mbox{cell}}}}{A}_{{{\mbox{cross,cell}}}})}{\sum {A}_{{{\mbox{cross,cell}}}}}$$

Finally, the number of droplets included in each parcel is obtained by dividing the total parcel volume by the volume of a single droplet.

The velocity of each parcel is assigned according to the velocity values stored in the map at the location where each given parcel is initialized. The turbulent kinetic energy and its dissipation rate are used to perturb the original direction of the stored velocity vectors, to account for the effect of turbulence-induced fluctuations^55^. As reported by Nocivelli et al.^56^, the initial perturbation of the parcel velocities is key to achieving accurate predictions of the interaction between the spray and the surrounding gas, the air entrainment, and the consequent initial plume expansion. Furthermore, the considerable difference in minimum grid size between the simulations of the internal flow (5–10 µm) and those of the Lagrangian-Eulerian spray (350 µm), along with the lack of gaseous mixture initialization at the orifice exits, can generate a sudden smoothing and reduction of the local turbulent kinetic energy, if compared to the values computed in the Eulerian-Eulerian calculations. This would unavoidably limit the downstream perturbing effect associated with the local turbulent dispersion, therefore requiring the initial perturbation at the time of parcel initialization.

In case the instantaneous simulation time falls between the timestamps of two consecutive maps or if the simulation time-step is shorter than the time resolution of the map file, the one-way coupling algorithm operates a time-based linear interpolation between the maps immediately before and after the current time-step to calculate quantities, such as the instantaneous velocity and temperature, or estimate the mass flow rate and injected mass. Hence, a finely time-resolved map file allows for more accurate representation of the transient events occurring during the injection^55,57^. In the current study, the maps were generated every 1 × 10^−7^ s to ensure a fine enough resolution as the minimum time-step observed during the injection was never lower than 5 × 10^−7^ s. More details about the one-way coupling model implementation can be found in previous publications^55,57^.

### Engine Specifications

The engine geometry used in this work is the result of a design optimization study previously published by Pei et al.^25^. In that work, a CFD-guided combustion system optimization was conducted using a RON80 gasoline for a heavy-duty compression-ignition engine targeting an engine-out NO_x_ level between 1.0 and 1.5 g kWh^−1^. A model year 2013 production Cummins ISX 15 diesel engine was used as the experimental platform to evaluate the performance of gasoline range fuels under mixing-controlled and low-temperature combustion conditions. The optimization was achieved via a design of experiments methodology specifically developed for engine combustion system design and optimization. Over 3000 simulations were performed on Argonne’s supercomputer Mira^58^ using a two-stage design of experiments in which the investigated parameters were piston bowl geometry (first stage) and spray targeting, fuel injection strategies, and swirl motion (second stage). It was found that the optimum bowl shape was a function of the operating conditions. For low-speed and low-load conditions, narrower designs allowed for better air utilization in the central portion of the combustion chamber, while wider designs were more suitable for medium-to-high speed and load conditions because of lower heat transfer through the bowl lip. The final design (i.e., the one employed in the current work) allowed for an estimated efficiency improvement of up to 6.3% at B50 condition and 5.1% at B25 condition, with the latter being the operating condition explored in the engine simulations performed in this work. The engine specifications relevant to the optimized piston bowl used in this study are listed in Table 1, where the Engine ratings are referred to the original design of the engine. More details about the optimization study are reported in the work of Pei et al.^25^.

**Table 1:** Engine specifications^25^.

Displacement volume	14.95 L
Number of cylinders	6
Bore	137 mm
Stroke	169 mm
Connecting rod length	262 mm
Compression ratio	
Original design	15.7
Optimized design	16.5
Fuel injection system	2500 bar common rail
Rotation speed	1375 rpm
Air system	single-stage variable geometry turbocharger high-pressure cooled exhaust gas recirculationcharge air cooler
Engine ratings	Power: 336 kW @ 1800 rpm Torque: 2373 N m @ 1000 rpm

The Engine ratings are referred to the original design of the engine.

**Fig. 9:**
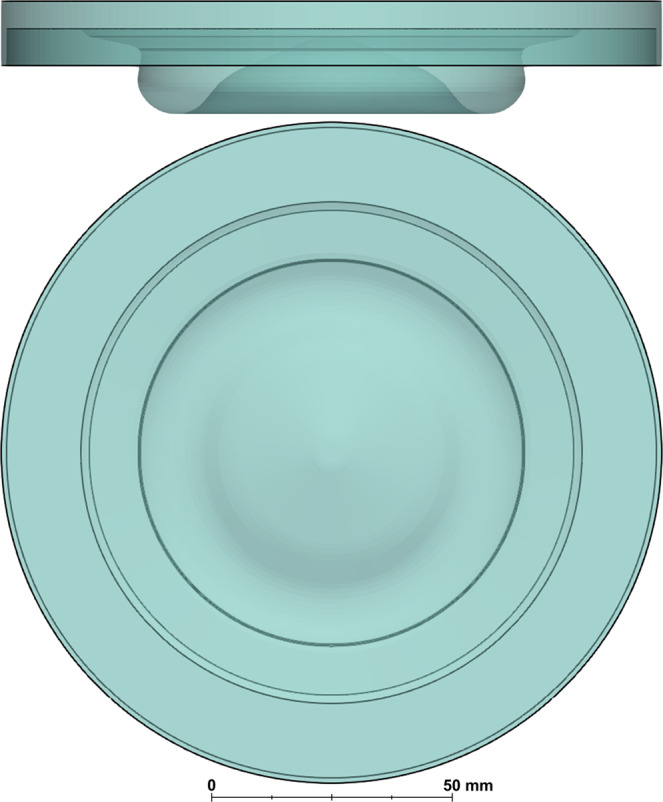
Side and top view of the engine geometry used in this work. The piston bowl geometry is the result of a design optimization carried out in previous work by Pei et al.^25^.

All the simulations performed in the work of Pei et al.^25^ were carried out using a sector mesh and a closed-cycle strategy, i.e., only the time interval between inlet valve closing and exhaust valve opening was simulated. In the current study, the geometry was modified to account for the whole extent of the combustion chamber in order to assess the effect of hole-to-hole spray variability on the combustion characteristics (cf. Figure 9). Consistent with the work of Pei et al.^25^, a flat cylinder head was employed. The authors are aware of the implications that this geometric simplification might have on the near-head flowfield development and plan to explore more realistic engine configurations in future studies. Nevertheless, there is good confidence that the considerations presented in this manuscript with regard to the spray-induced variability will hold their validity even when using a simplified head geometry such as the one simulated in this work.

## Data Availability

All the data used to support the findings of this study can be made available by the corresponding authors upon reasonable request.
